# Type 2 diabetes mellitus plays a protective role against osteoporosis --mendelian randomization analysis

**DOI:** 10.1186/s12891-023-06528-1

**Published:** 2023-06-02

**Authors:** Lulu Cheng, Siyu Wang, Hailan Tang

**Affiliations:** 1 Graduate School, Wuhan Sports University, Wuhan, 430079 Hubei China; 2 College of Acupuncture-Moxibustion and Tuina, Anhui University of Chinese Medicine, Hefei, 230012 Anhui China

**Keywords:** Type 2 diabetes mellitus, Osteoporosis, Mendelian randomization

## Abstract

**Background:**

Type 2 diabetes mellitus (DM2) and osteoporosis (OP) are currently the two most significant causes of mortality and morbidity in older adults, according to clinical evidence. The intrinsic link between them is yet unknown, despite reports of their coexistence. By utilizing the two-sample Mendelian randomization (MR) approach, we sought to evaluate the causal impact of DM2 on OP.

**Methods:**

The aggregate data of the whole gene-wide association study (GWAS) were analyzed. A two-sample MR analysis was performed using single-nucleotide polymorphisms (SNPs), which are strongly associated with DM2, as instrumental variables (IVs) to evaluate the causal analysis of DM2 on OP risk with OR values, using inverse variance weighting, MR-egger regression, and weighted median methods, respectively.

**Result:**

A total of 38 single nucleotide polymorphisms were included as tool variables. According to the results of inverse variance-weighted (IVW), we found that there was a causal relationship between DM2 and OP, in which DM2 had a protective effect on OP. For each additional case of DM2, there is a 0.15% decrease in the odds of developing OP (OR = 0.9985;95%confidence interval:0.9974,0.9995; *P* value = 0.0056). There was no evidence that the observed causal effect between DM2 and the risk of OP was affected by genetic pleiotropy (*P* = 0.299). Using Cochran Q statistics and MR-Egger regression in the IVW approach, the heterogeneity was calculated; *P* > 0.05 shows that there is a significant amount of heterogeneity.

**Conclusion:**

A causal link between DM2 and OP was established by MR analysis, which also revealed that DM2 decreased the occurrence of OP.

**Supplementary Information:**

The online version contains supplementary material available at 10.1186/s12891-023-06528-1.

## Introduction

Osteoporosis(OP) is a disease characterized by reduced bone strength, mainly associated with bone tissue loss and bone microstructure destruction, which makes patients highly susceptible to fractures and substantially reduces their quality of life [1]. The early clinical symptoms of OP are pain and limitation of movement, which can lead to skeletal deformities and fractures in severe cases [2]. Clinical causes of OP include gender, age, hypogonadism, various diseases, and medications. Among them, disease factors include gastrointestinal diseases, rheumatic immune diseases, endocrine diseases, kidney diseases, etc. Diabetes mellitus is a common disease of the endocrine system, and its incidence is increasing year by year. Moreover, osteoporosis caused by diabetes is becoming more common and of increasing attention [3]. Hyperglycemia in diabetic patients can cause a variety of chronic complications. OP is one of the most common complications, which can cause joint dysfunction, long-term pain, and a high risk of disability [4]. Diabetes and OP are both common chronic diseases. The link between the two was proposed by Albright as early as 1948 [5]. There is a consensus that Type 1 diabetes mellitus (DM1) increases the risk of osteoporosis. The mechanism of osteoporosis in combination with DM1 is clear, and researchers generally believe that insulin deficiency interferes with bone formation and mineralization and accelerates bone resorption. At the same time, most DM1 patients develop in adolescence, with incomplete bone mineralization and low peak bone mineral density, thus increasing the degree of bone loss [6]. The relationship between DM2 and the pathogenesis of OP is still controversial, as the pathogenesis of DM2 is still not fully understood and its effect on bone metabolism is different compared to DM1 and can be influenced by a variety of other factors. According to the investigation material, the prevalence of diabetic osteoporosis patients is up to more than 50%, while some studies have found inconsistent results in the relationship between DM2 patients and bone mineral density (BMD), which can be normal, increased, or decreased [7, 8]. The intricacies of many factors have brought great interference to the study of the relationship between DM2 and OP.

In epidemiological studies, the presence of mixed factors has greatly interfered with the causal inference of exposure and outcome. Mendelian randomization (MR) studies are a novel approach to genetic analysis that reduces the effects of confounding, based on the principle that genetic alleles are naturally randomly distributed in a population. It mainly analyzes the genetic variation representing the exposure factors and the outcome events and then explores whether there is a causal relationship between the exposure factors and the outcome events [9, 10]. Unlike traditional observational studies, which are susceptible to reverse causality, MR studies have genetic variations that are determined long before the embryo is formed and thus are not susceptible to acquired diseases or other confounding factors. MR Avoids some of the limitations of observational studies (confounding, reverse causality, regression dilution bias) and RCTs (randomized controlled trials) in making causal inferences [11]. With the discovery of a large number of genetic variants in biology that are strongly associated with specific traits and the public release of hundreds of thousands of aggregated data on the association of exposures and diseases with genetic variants from many large sample genome-wide association studies (GWAS), these aggregated data have allowed researchers to estimate genetic associations in large sample data [12]. Therefore, we hope to investigate the potential impact of DM2 on OP by using MR to elucidate it at the genetic level.

## Methods

No further ethics approval was required because the already gathered and published data were used in this reanalysis.

### Exposure GWAS-DM2

**Fig. 1:**
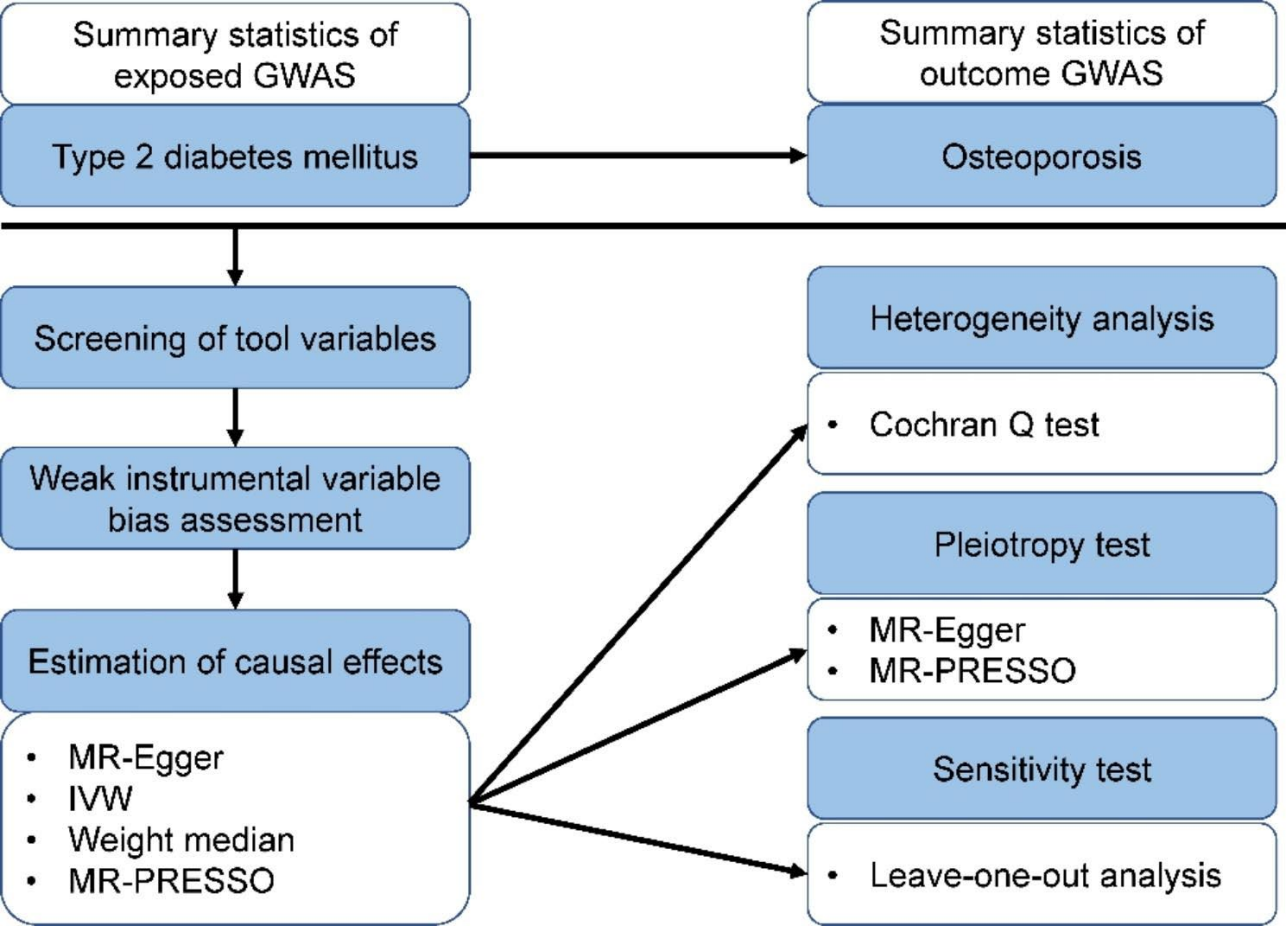
A two-sample Mendelian randomization analysis of the causal association between Type 2 diabetes mellitus and osteoporosis. GWAS, genome-wide association studies; MR, Mendelian randomization; IVW, inverse variance weighting

DM2 served as the study’s exposure factor, and OP served as the study’s outcome variable. Gender heterogeneity was assessed using the Cochran Q test, and causal association analysis was carried out using the two-sample MR Analysis. Sensitivity analysis was done to verify that the results of the causal connection were accurate. Figure 1 shows an overview of the study’s design. GWAS for osteoporosis (OP) and estimated DM2 were retrieved from https://gwas.mrcieu.ac.uk/datasets. The summary results of a recent GWAS meta-analysis on DM served as the basis for the main genetic tools (Table 1). The meta-analysis examined 29,166 DM2 patients and 183,185 controls in total. To minimize the impact caused by linkage disequilibrium (LD), we set the threshold of statistical significance as “P < 5 × 10 − 8; LD r^2^ < 0.0001, kb = 10000” to identify the SNPs associated with DM2 [13]. To compensate for the deletion, SNPs with strong LD (r^2^ > 0.8) with the missing SNPs were used, and SNPs with no replacement site were excluded.The heterogeneity test was used to eliminate the significantly heterogeneous SNPs, and finally 38 SNPs significantly associated with DM2 were obtained as instrumental variables.

**Table 1:** Details of Studies and Datasets Used in the Study

Exposure /outcomes	ID	Sample size	SNP size	Year	Population Studied	Web source
**Type 2 diabetes mellitus**	finn-b-E4_DM2_STRICT	212,351	16,380,434	2021	European/males and females	https://gwas.mrcieu.ac.uk/datasets/finn-b-E4_DM2_STRICT/
**Osteoporosis**	ukb-b-12,141	462,933	9,851,867	2018	European/males and females	https://gwas.mrcieu.ac.uk/datasets/ukb-b-12141/

### Outcomes in GWAS: OP

OP data were derived from 7547 OP samples and 455,386 control samples in UK biobank. The UK Biobank is a large-scale constructive cohort study that collects samples from about 500,000 UK residents ranging in age from 37 to 76. Details of all GWASs included in our study are represented in Table 1.

### Statistical analyses

All MR analyses were performed using the “TwoSampleMR”, “MendelianRandomization” and “MR_presso” packages in the R software (version 4.1.2 with packages, R Foundation for Statistical Computing, Vienna, Austria).In this study, inverse variance weighted (IVW) [14], MR-Egger regression [15], and weighted median estimator ( WME ) [16] were used for MR analysis. The IVW principle is to weigh the inverse of the variance of each IV as the weight while ensuring that all IVs are valid, the regression does not consider the intercept term, and the final result is the weighted average of the effect values of all IVs. The major difference between the MR - Egger method and IVW is that the regression takes into account the presence of the intercept term, and also it uses the inverse of the ending variance as a weight for the fit. WME is defined as the median of the weighted empirical density function of the ratio estimates, which allows consistent estimation of causality if at least half of the valid instruments in the analysis are available.

### Sensitivity analysis

The heterogeneity test [17] is mainly to test the difference between individual IVs, if the difference between different IVs is large, then the heterogeneity of these IVs is large, this study uses the random effects model to estimate the MR effect; the Pleiotropy test [18] is mainly to test whether multiple IVs have horizontal pleiotropy. The intercept term of the MR-Egger method is commonly used to indicate the presence of horizontal pleiotropy if this intercept term is significantly different from 0 [19]. The leave-one-out sensitivity test is mainly to calculate the MR Results of the remaining IVs after the removal of IVs one by one [20]. If the MR results estimated by other IVs after the removal of a certain IV are very different from the total results, it indicates that MR Results are sensitive to the IV.

## Result

### Instrumental variables for mendelian randomization

There were 38 SNPs in total after IVs with linkage disequilibrium were excluded. Table 2 provides a summary of SNPs’ fundamental data. The distribution range of F statistics corresponding to a single SNP is 371.661 ~ 6516.944, which suggests that weak instrumental variable bias is less likely to have an impact on causal connection.

**Table 2:** Characteristics of the SNPS Associated with DM2 and their Association with OP

SNPs	CHR	Gene	EA	EAF	DM2			OP		
					β	SE	*P*	β	SE	*P*
**rs10245867**	7:28142186	JAZF1	T	0.3307	0.0666	0.0115	6.55E-09	-9.02E-05	0.000279	7.50E-01
**rs1046317**	4:6304242	WFS1	C	0.6105	0.0842	0.0111	3.96E-14	-8.58E-05	0.000281	7.60E-01
**rs10830963**	11:92708710	MTNR1B	G	0.3567	0.1316	0.0113	2.35E-31	-0.000521543	0.000293	7.50E-02
**rs10882099**	10:94460650	HHEX	C	0.4772	-0.0788	0.0108	3.41E-13	-4.88E-05	0.000267	8.50E-01
**rs10938397**	4:45182527	NMU	G	0.4736	0.0742	0.0108	7.52E-12	-0.000737957	0.000265	5.40E-03
**rs11257659**	10:12309269	/	T	0.2647	0.0854	0.0123	4.23E-12	-0.000210313	0.000337	5.30E-01
**rs11263763**	17:36103565	HNF1B	A	0.646	-0.0665	0.0113	4.31E-09	-1.08E-05	0.000264	9.70E-01
**rs112694524**	2:43453721	ZFP36L2	A	0.03348	-0.1807	0.0306	3.70E-09	-0.000573605	0.000503	2.50E-01
**rs1128249**	2:165528624	COBLL1	T	0.3478	-0.0705	0.0114	6.11E-10	0.00020851	0.000269	4.40E-01
**rs11558471**	8:118185733	SLC30A8	G	0.3785	-0.0814	0.0112	2.97E-13	-0.000280967	0.000282	3.20E-01
**rs11712037**	3:12344730	PPARG	G	0.1708	-0.1091	0.0144	3.76E-14	0.00089166	0.000405	2.80E-02
**rs12967878**	18:57826570	RP11-795H16.3	C	0.181	0.077	0.014	3.91E-08	-0.000804767	0.000313	1.00E-02
**rs1515110**	2:227122216	NEU2	T	0.6172	0.0764	0.0111	6.14E-12	-1.76E-05	0.000273	9.50E-01
**rs1798085**	12:71550697	TSPAN8	C	0.5575	-0.0598	0.0109	3.58E-08	-5.83E-05	0.000263	8.20E-01
**rs2303700**	19:7976529	MAP2K7	C	0.6741	-0.0675	0.0116	5.75E-09	4.27E-05	0.000284	8.80E-01
**rs2781655**	6:131881146	RP11-394G3.2	T	0.2218	0.0766	0.013	4.26E-09	0.000428793	0.000339	2.10E-01
**rs28642213**	9:139248082	GPSM1	G	0.6971	0.1004	0.0118	1.55E-17	-6.22E-05	0.000303	8.40E-01
**rs429358**	19:45411941	APOE	C	0.1827	-0.0813	0.0142	1.09E-08	-0.000157671	0.000363	6.60E-01
**rs5215**	11:17408630	KCNJ11	T	0.5286	-0.0619	0.0108	1.12E-08	9.91E-06	0.000274	9.70E-01
**rs55993634**	16:75236763	CTRB2	G	0.08714	-0.1646	0.0194	2.10E-17	-0.000196203	0.000483	6.80E-01
**rs56348580**	12:121432117	HNF1A	C	0.2829	-0.0785	0.0121	8.39E-11	-2.06E-05	0.000285	9.40E-01
**rs57307671**	7:102086605	ORAI2	T	0.1834	0.0996	0.014	1.21E-12	0.000322521	0.0004	4.20E-01
**rs62492368**	7:150537635	AOC1	A	0.3401	0.0768	0.0114	1.84E-11	-0.000499531	0.000286	8.10E-02
**rs6780171**	3:185503456	IGF2BP2	A	0.3063	0.094	0.0117	9.80E-16	-0.000482843	0.000283	8.80E-02
**rs6786846**	3:170629884	EIF5A2	A	0.681	0.0733	0.0116	2.88E-10	7.84E-05	0.000272	7.70E-01
**rs7018475**	9:22137685	CDKN2B-AS1	G	0.2788	0.1144	0.0121	3.17E-21	-0.000150302	0.0003	6.20E-01
**rs7109575**	11:72463435	ARAP1	A	0.2376	-0.094	0.0128	1.81E-13	0.000142506	0.000362	6.90E-01
**rs71330995**	3:123124513	ADCY5	A	0.1879	-0.0928	0.0139	2.46E-11	0.000296116	0.000295	3.20E-01
**rs7224685**	17:4014384	ZZEF1	T	0.3016	0.0644	0.0117	4.19E-08	-0.000290586	0.000282	3.00E-01
**rs7451008**	6:20673880	CDKAL1	C	0.3292	0.128	0.0115	6.31E-29	-0.000253833	0.000298	3.90E-01
**rs745805**	4:185718132	ACSL1	T	0.1503	-0.083	0.0152	4.64E-08	-0.000221746	0.000345	5.20E-01
**rs76177300**	5:102143311	PAM	A	0.05776	0.1386	0.0232	2.29E-09	-0.000442024	0.000589	4.50E-01
**rs7903146**	10:114758349	TCF7L2	T	0.1992	0.3055	0.0138	3.03E-109	-6.57E-05	0.000289	8.20E-01
**rs7998259**	13:80718654	RP11-470M1.2	A	0.3896	-0.0769	0.0112	5.56E-12	0.000694742	0.000277	1.20E-02
**rs8353**	22:20796117	KLHL22	T	0.3009	-0.0744	0.0118	2.94E-10	0.000419189	0.000293	1.50E-01
**rs878521**	7:44255643	CAMK2B	A	0.2072	0.0894	0.0133	2.05E-11	0.000112942	0.000303	7.10E-01
**rs9505086**	6:7232186	RREB1	C	0.4439	0.064	0.011	5.12E-09	0.000214134	0.00027	4.30E-01
**rs9933509**	16:53818167	FTO	C	0.4126	0.1185	0.011	3.53E-27	-0.000910019	0.000266	6.30E-04

SNP, single nucleotide polymorphism; CHR, Chromosomes; EA, effect allele; EAF, the effect allele frequency; β, Allelic effect value; SE, standard error

### Mendelian randomization between DM2 and OP

**Fig. 2:**
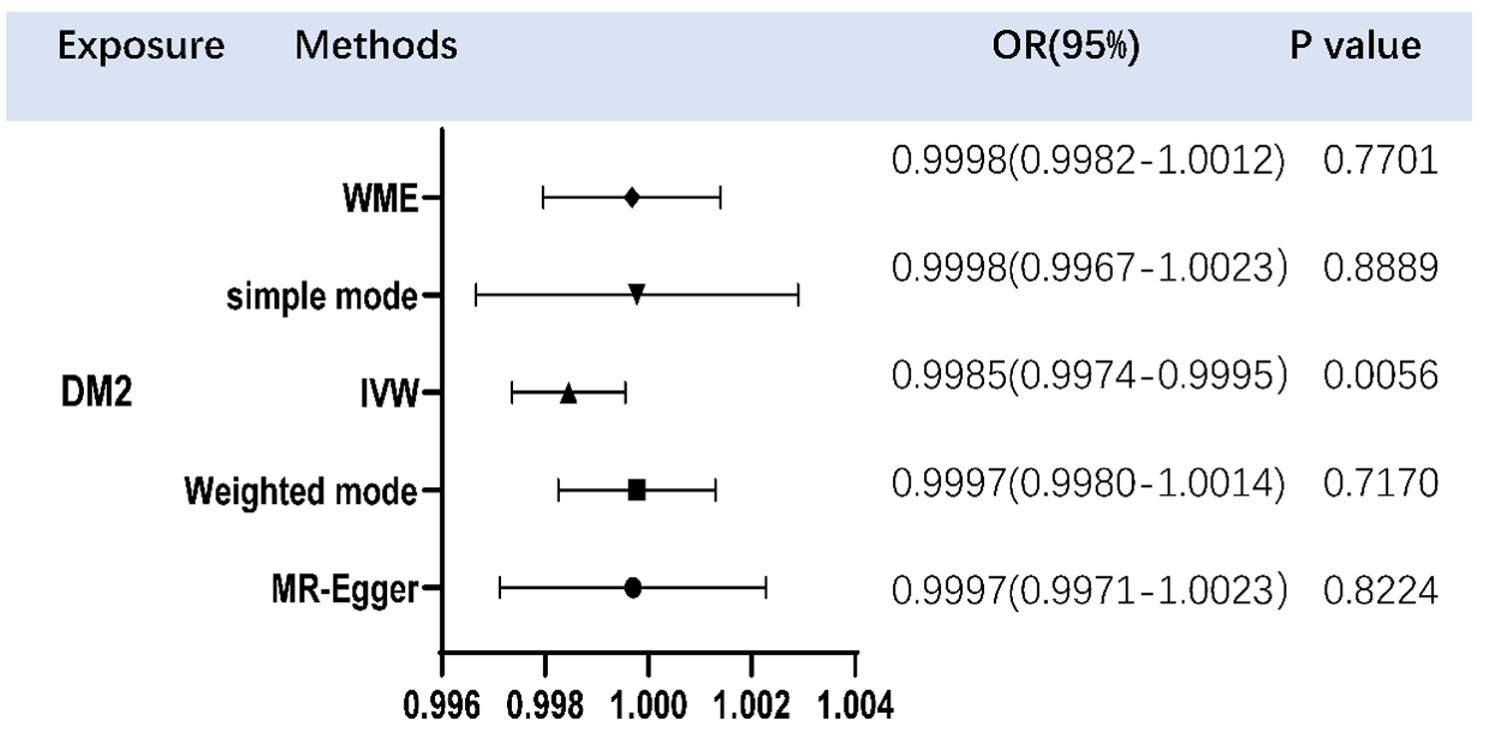
The OR values of IVW, MR-Egger regression, and WME and their 95% CI (confidence interval). The different shapes in the figure represent the OR corresponding to the use of different measurement methods. DM2, Type 2 diabetes mellitus; WME, weighted median estimator; IVW, inverse variance weighted; OR, Odds ratio; P value, P value of the causal estimate

**Fig. 3:**
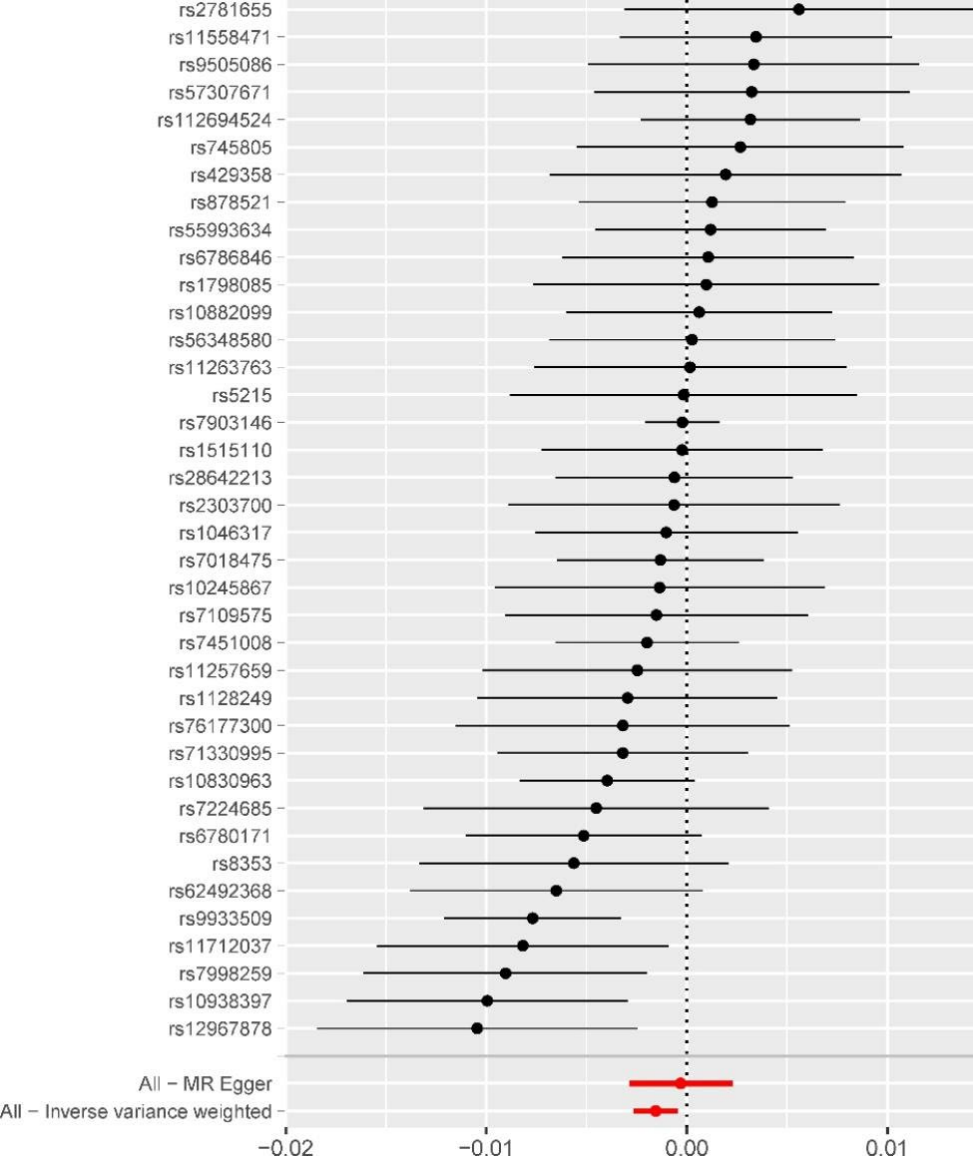
Forest plot for single nucleotide polymorphisms associated with Type 2 diabetes mellitus and osteoporosis. For each of the 38 significant non-pleiotropic Type 2 diabetes mellitus SNPs, the forest plot shows the estimate of the effect of genetically-increased Type 2 diabetes mellitus risk on osteoporosis, as assessed for each SNP, the 95% confidence intervals (indicated with black lines)

**Fig. 4:**
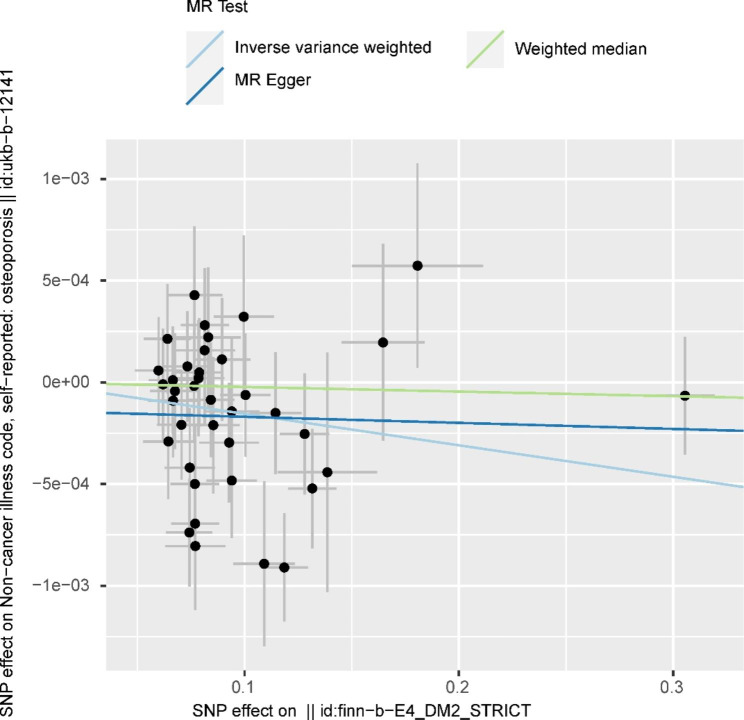
Scatterplots of the causal relationships between Type 2 diabetes mellitus and osteoporosis. The X-axis represents the genetic tool-Type 2 diabetes mellitus association and the Y-axis represents the genetic tool-osteoporosis association. The slope of each line corresponds to the estimated MR effect of each method

There is a negative correlation between DM2 and OP at the level of genetic prediction, according to Fig. 2’s MR estimates of the various methods used to evaluate the causative effects of DM2 on OP (IVW: OR = 0.9985, P value = 0.0056, 95%confidence interval (CI):0.9974,0.9995). The outcomes of the weighted mode, WME, simple mode, and MR-Egger, however, were not statistically significant (Fig. 2). These results could also be observed in the forest plot (Fig. 3) and the scatter diagram (Fig. 4).

### Heterogeneity and sensitivity tests

**Fig. 5:**
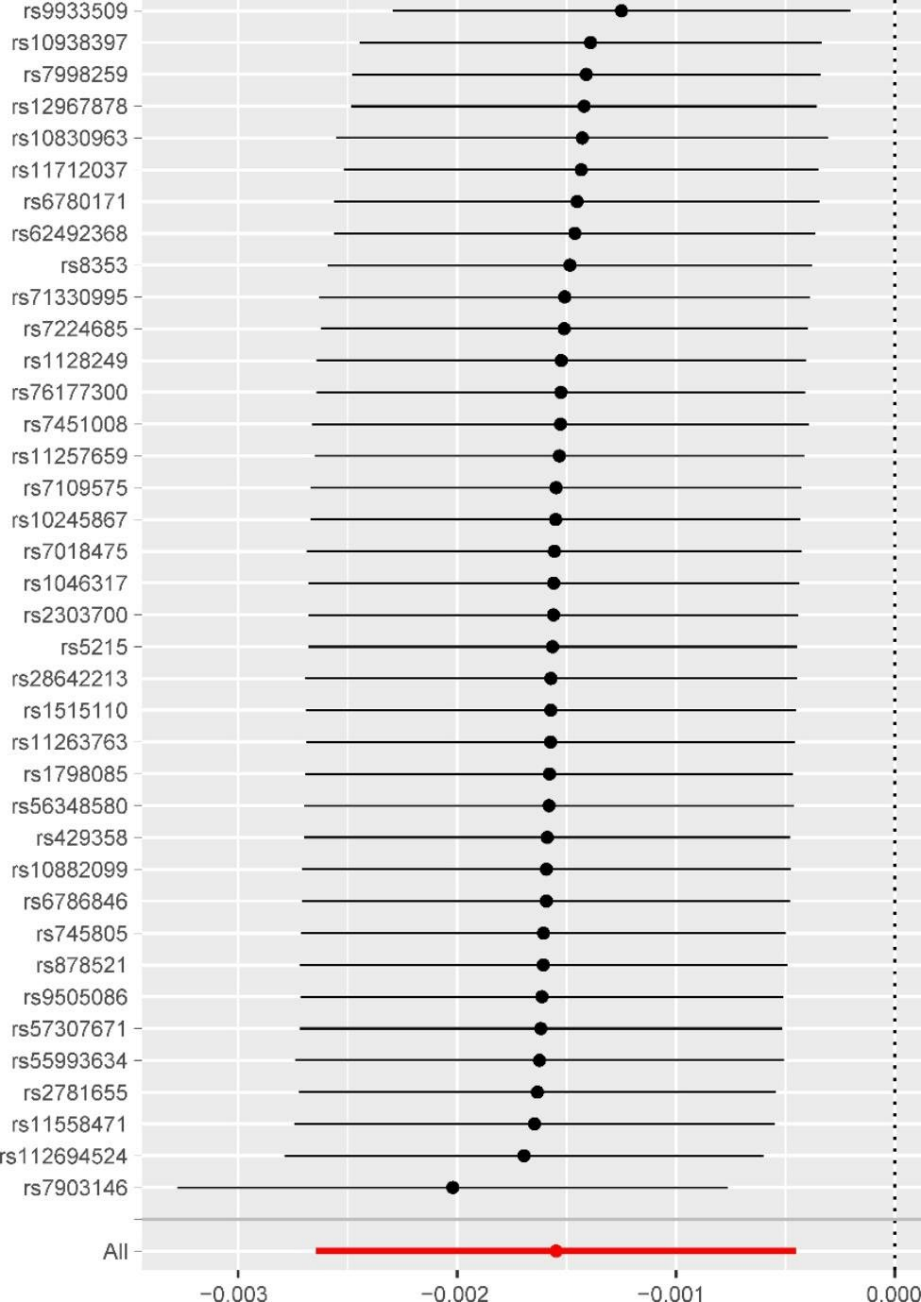
Leave-one-out of single nucleotide polymorphisms associated with Type 2 diabetes mellitus and osteoporosis

SNPs were not heterogeneous, according to the Cochran Q test for IVW (*P* = 0. 08) and MR-Egger regression (*P* = 0. 08). As no statistically significant difference between the egger_intercept of MR-Egger and 0 (*P* = 0. 30), we can infer the presence of no horizontal pleiotropy in SNPs. When a single SNP is used as IV, the funnel plot displays a symmetric distribution of dots representing causal association effects, indicating that causal linkages are less likely to be influenced by possible bias (Figure S1). The results of the leave-one-out sensitivity analysis revealed that, after removing each SNP in turn, the IVW analysis results of the remaining 37 SNPs were identical to those of the analysis results of all SNPs included (Fig. 5), and no SNPs that significantly affected the estimates of the causal association were discovered. On the basis of the IVW, horizontal pleiotropy test, retention technique analysis, and other findings, we concluded that the genetically predicted DM2 is the protective factor of OP.

## Discussion

A protective causal relationship between DM2 and OP was found by MR analysis of both samples based on published data and a large-scale GWAS study. For each additional case of DM2, there is a 0.15% decrease in the odds of developing OP(OR=0.9985;95%CI:0.9974,0.9995; *P* value = 0.0056). In practical terms, it means that while there might be a slight protective effect of DM2 on OP, it is so small that it would likely have minimal clinical significance or impact on individuals’ risk of developing OP.OP is an age-related metabolic bone disease, the acceleration of the aging process has made OP increasingly a major issue affecting people’s health [21]. DM2 has become a health hazard worldwide. With the continuous growth of population aging, the prevalence of DM2 has increased significantly [22]. DM2 constitutes 8.8% of the population all over the world and its frequency is gradually increasing [23]. The causes of OP resulting from diabetes are intricate [24]. Abnormal glucose metabolism in diabetic patients can affect bone metabolism through various pathways, which in turn can lead to OP. People with DM1 are more prone to osteoporosis while data for DM2 is emerging [25]. The etiology of DM2 and OP is affected by multiple factors such as environment and genetics, and many genes are involved [26].

The currently accepted gold standard for the diagnosis of OP is BMD detected by Dual-energy X-ray absorptiometry [27]. With the increase of bone fragility in DM patients, the risk of fracture is significantly increased [28], DM1 can significantly increase the risk of fracture, and the BMD is usually decreased. OP induced by DM2 is manifested in decreased bone strength, and many studies have found that most of the BMD is normal or elevated [7]. A meta-analysis including 35 studies showed that hip and lumbar spine BMD was 4–5% higher in patients with DM2 (Mexican Americans, whites, and blacks) than in nondiabetic individuals, which may be partly explained by the predominance of overweight and obesity in patients with DM2 [29]. It has been shown that early hyperinsulinemia and body fat content in patients with DM2 may be associated with increased BMD [30]. The studies also demonstrated increased bone formation in Older patients with DM2, characterized by the increased bone formation and an increase in BMD that may be associated with the observed increase in bone formation, while bone resorption is driven the by Parathyroid hormone [31]. Studies in the East Asian population have shown a weak association between DM2 and increased BMD, but some studies have shown that long duration, low body mass index (BMI), smoking, and chronic complications are risk factors for decreased BMD in patients with DM2 [32]. So far, numerous studies on the correlation between DM2 and OP have elaborated on the relationship between them, but they do not explain the causal relationship well due to the interference of numerous confounding factors. The present study used genetic variation as an instrumental variable to greatly exclude confounding by acquired confounders and to strengthen the evidence for a causal increase in the protective effect of DM2 on OP.

Related studies have demonstrated the mechanism and principle of action betweenDM2 and OP, providing theoretical support for this study. The fact that diabetes status was a significant predictor of BMD in women independently of BMI may be partly explained by the anabolic effect that insulin has on bone tissue. Since type 2 diabetes is preceded by a period of insulin resistance, hyperinsulinemia may confer a protective effect on BMD, either directly through elevated fasting insulin as demonstrated in diabetic and non-diabetic elderly men and women [33] or indirectly through BMI [34]. Some studies have shown the prevalence of overweight or obese status in DM2 patients. Obesity requires greater mechanical loading on the skeleton and reactively causes an increase in BMD to adapt to the greater load [35], in addition, adipose tissue is the main source of estrogen in postmenopausal women, and estrogen increases bone mass by inhibiting osteoclast activity; therefore, obesity is thought to be positively associated with BMD and has a protective effect on OP [36, 37]. The mechanism is that peripheral adipose tissue can promote the conversion of androgens into estradiol and androstenedione into estrone, while leptin and lipocalin formed in the process of obesity inhibit the level of sex hormones and globulin, so that the level of free sex hormones increases, and a certain concentration of estrogen has a protective effect on bone, and estradiol and estrone can reduce bone resorption [38]. Most cross-sectional studies using DXA demonstrated higher or normal BMD in DM2 patients [39]. Increased BMD Z-scores in DM2 patients in the spine (0.41 ± 0.01) and hip (0.27 ± 0.01), when compared to non-diabetic people, were reported by pooled estimates from a meta-analysis of significant studies [40]. Although obesity and overweight are linked to DM2, which increases BMD, the aforementioned discrepancies persisted even after body size was taken into consideration in the majority of studies. Even after controlling for confounding variables like body weight, the majority of large-scale epidemiological studies show normal or above-normal BMD, with T-scores 0.5 higher in patients with DM2 than in healthy controls, and more in women than in men [41]. Conversely, DM2 patients frequently have greater BMIs, therefore it may be anticipated that they are at decreased risk. All of these studies suggest that DM2 has a positive effect on OP and may reduce the risk of OP. Once OP occurs, it cannot be reversed, so early prevention is especially critical. In summary, the association between DM2 and OP has been confirmed by both traditional observational epidemiological studies and basic studies, but the causal association between the two is still controversial. In this study, we propose to investigate the causal association between DM2 and OP using the MR method, taking into account the principles and conditions of the MR method.

This is a study that uses MR models to explore the causal association between DM2 and OP. First, the application of the MR model controls the effects of confounding factors and inverse results on the estimation, thus obtaining reliable estimates of causal effects based on observational studies [42].In addition, the aggregated data MR model uses GWAS based on large samples as the dataset, and the inclusion of large samples largely improves the test performance compared to the small sample model based on individual data [10]. Second, the Generalized Summary data-based MR approach has higher test efficacy than other MR models because it controls for both instrumental and exposure and outcome effect errors, and corrects for the biasing effect of LD between instrumental variables on the results [43]. Finally, MR methods are less likely to be biased by confounding factors or reverse causality than traditional observational studies, so our results provide more convincing evidence in support of a causal relationship between DM2 and OP.

This study has some limitations. First, Genetic polymorphisms are difficult to verify, and even if we use the MR Egger method, we cannot completely rule out the misclassification of genetic polymorphisms. In our data analysis, some of the methods we used did not yield the same conclusions as IVW due to the univariate analysis, but the SNPS included in this study met the hypothesis of being effective instrumental variables. The demand for consistent beta direction across all MR approaches has been enhanced in the majority of MR analyses, which was also the case in our study [44]. Second, the SNPs used are from the European population, which may lead to bias. It is unclear whether the results can be directly applied to other populations, and more comprehensive studies between different ethnic groups should follow. Third, MR analysis was used to determine the causal relationship between DM2 and OP risk. Because single nucleotide polymorphisms may also be potentially associated with confounding factors, such as BMI, MR analyses based on genome-wide association analysis data may overestimate the association between genetics and exposure. Furthermore, further biological studies and randomized controlled trials are needed to validate the findings of this study and to support the biological role of DM2 in the pathogenesis of OP.

## Conclusion

This study found that DM2 can reduce the incidence of OP. These findings explain the causal relationship between OP and OA from a genetic perspective and provide new insights into the future relationship between OP and OA. Due to the limitations of MR analysis and the very weak protective effect of DM2 on OP, a large number of RCTS are needed to further verify whether there is a potential cause-and-effect relationship between DM2 and OP.

## Electronic supplementary material

Below is the link to the electronic supplementary material.

**Supporting Information: FIGURE S1** Funnel plots to the causal association of Type 2 diabetes mellitus on osteoporosis.

## Data Availability

The datasets analyzed during the current study are available in the open gwas repository, [https://gwas.mrcieu.ac.uk/datasets].
