# Supplementary material for: Type 2 diabetes mellitus plays a protective role against osteoporosis --mendelian randomization analysis
Source: BMC Musculoskelet Disord. 2023 Jun 2;24:444. doi: 10.1186/s12891-023-06528-1 (PMC10236750; doi:10.1186/s12891-023-06528-1)
Supplement: Supplementary file 1 — Supporting Information: FIGURE S1 Funnel plots to the causal association of Type 2 diabetes mellitus on osteoporosis. [file 12891_2023_6528_MOESM1_ESM.docx]

**Supporting Information**

**Type 2 diabetes mellitus plays a protective role against osteoporosis --****Mendelian randomization analysis**

**Lulu Cheng^1,2*^,Siyu Wang ^1^,Hailan Tang^1^**

*Correspondence:chenglulu958@163.com

1. Wuhan Sports University,430079 Wuhan,Hubei,P.R. China.

2. Anhui University of Chinese Medicine,230012,Hefei Anhui,P.R. China.

**Contents**

**1. Supporting figure**


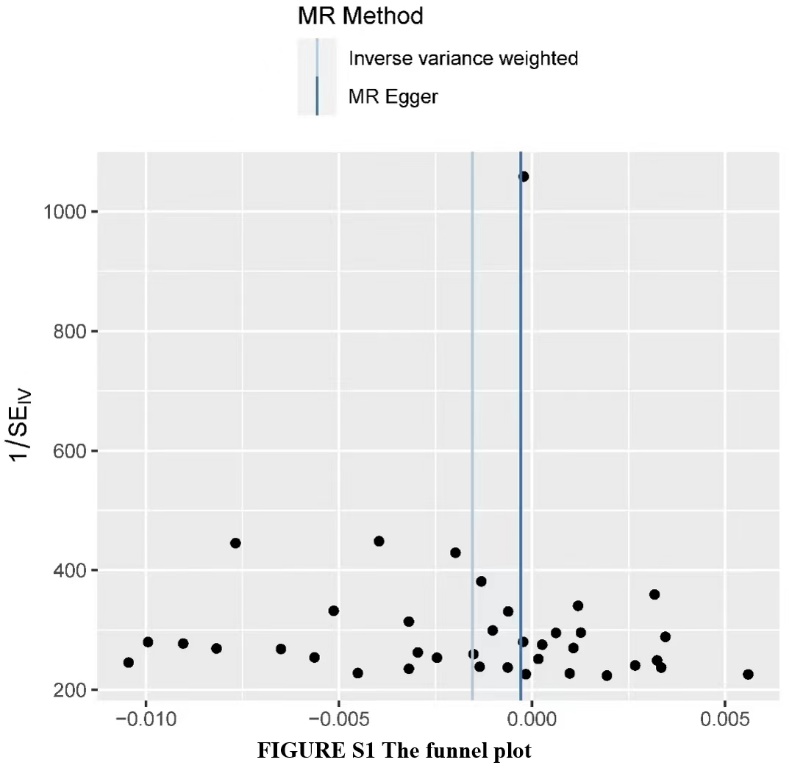


FIGURE S1 Funnel plots to the causal association of Type 2 diabetes mellitus on osteoporosis
